# A Multi-Phase Based Multi-Application Mapping Approach for Many-Core Networks-on-Chip

**DOI:** 10.3390/mi12060613

**Published:** 2021-05-26

**Authors:** Fen Ge, Chenchen Cui, Fang Zhou, Ning Wu

**Affiliations:** College of Electronic and Information Engineering, Nanjing University of Aeronautics and Astronautics, Nanjing 211106, China; cccwade@nuaa.edu.cn (C.C.); zfnuaa@nuaa.edu.cn (F.Z.); wunee@nuaa.edu.cn (N.W.)

**Keywords:** networks-on-chip, multi-application, mapping

## Abstract

More and more attention is being paid to the use of massive parallel computing performed on many-core Networks-on-Chip (NoC) in order to accelerate performance. Simultaneously deploying multiple applications on NoC is one feasible way to achieve this. In this paper, we propose a multi-phase-based multi-application mapping approach for NoC design. Our approach began with a rectangle analysis, which offered several potential regions for application. Then we mapped all tasks of the application into these potential regions using a genetic algorithm, and identified the one which exhibited the strongest performance. When the packeted regions for each application were identified, a B*Tree-based simulated annealing algorithm was used to generate the optimal placement for the multi-application mapping regions. The experiment results show that the proposed approach can achieve a considerable reduction in network power consumption (up to 23.45%) and latency (up to 24.42%) for a given set of applications.

## 1. Introduction

Due to the advancement of transistor technology, hundreds to thousands of processors or cores have now been integrated on a single chip. Networks-on-Chip (NoC) has emerged as an efficient and scalable interconnect solution to address the challenges of the increasing concurrent communication requirements in such many-core processor systems [1,2]. Massive parallel computing performed on many-core NoC is the present and future of computing [1,3]. To realize higher level parallelism, it is no longer reasonable to focus only on the implementation of single applications, given the abundant processing elements available on many-core NoC. Multiple applications could be deployed on different regions of the NoC and executed in parallel [4].

Based on the existing NoC platforms, much research addressing the application mapping problem has been undertaken in recent years. However, much of this research has been carried out using single-application mapping [5,6,7,8,9,10], and only a few multi-application mapping methods were proposed. Murali et al. first presented a methodology to map multiple use-cases onto NoC architecture, satisfying the constraints of each use-case [11]. A multi-objective adaptive immune algorithm which considered different various delay constraints for multi-application mapping was proposed in [12]. In these studies, multiple applications reused the same platforms in different time slots. The main drawbacks of these systems were the time overhead incurred by reconfiguring the NoC and loading new applications, and the fact that multiple applications were not considered to execute in parallel. Yang et al. [4] proposed a multi-application mapping method to identify an optimal mapping region for each application. However, it dealt with multiple applications sequentially on a fixed platform. The applications mapped later may have fewer choices, which is not equitable for these applications. A fault-tolerant multi-application mapping algorithm was proposed by Khalili et al. [13]. The main goal of the algorithm was to map an application to free non-faulty processing cores and identify the best spare core placements. Zhu et al. [14] proposed an efficient heuristic-based algorithm for solving the issue of balancing minimized on-chip packet latency with performance-awareness in the multi-application mapping of chip-multiprocessors. Khasanov et al. [15] proposed an algorithm to map applications on heterogeneous multicore systems. However, all these studies focused on the scheduling of threads or tasks in each application.

In this paper, we propose a multi-phase-based multi-application mapping approach for NoC design analogous to those used in [4], but completely different in its algorithm design. Our approach began with a rectangle analysis which identified several potential regions for an application. Then we mapped all tasks of the application into these potential regions and identified the one which exhibited the strongest performance. When the packeted regions for each application were identified, a B*Tree-based simulated annealing (SA) algorithm was used to generate the optimal placement of the mapping regions. The aim of our proposed approach is to identify the best performance for each individual application mapping, then packet each application mapping region as a block to determine placements for all the applications with a minimized mapping area of the NoC platform.

The rest of the paper is organized as follows. Section 2 describes problem formulation and definitions. Section 3 presents our multi-application mapping approach. The results of our experiment are demonstrated in Section 4, and we conclude our work in Section 5.

## 2. Problem Formulation and Definitions

In the single-application scenarios, the mapping problem is how to identify an appropriate position for each task of the application according to particular performance or cost metrics. In the multi-application scenarios, the problem is extended to the search for the optimal positions for both the applications and tasks of the individual application [4]. In order to formulate this mapping problem, we require the following definitions.

**Definition** **1.** *A single application can be denoted by a task digraph TG(T,A). Each vertex t_i_* *∈* *T represents a task, and each edge a_i,j_* *∈* *A represents the communication from task t_i_ to t_j_. Every edge has one attribute, denoted by v_i,j_, which represents the total volume of communication. Multiple applications can be represented by a set of applications S = {TG_1_,TG_2_, … TG_n_}, where n is the number of the given applications.*

**Definition** **2.** *T* *he target architecture NoC composed of n cores is modeled by an architecture graph NAG (C,L), where C = {c_1_,c_2_, … c_m_} is the set of cores in the NoC platform and L is the set of links between cores. In this paper, the NoC is assumed to be a homogeneous 2D architecture using a deterministic XY routing algorithm.*

**Definition** **3.** *R_i_ denotes the mapping region on the NAG for application TG_i_. P = {(x_i_,y_i_)|1 ≤ I ≤ n} is an assignment of the rectangular regions R_i_’s with the coordinates of their bottom-left corners being assigned to (x_i_,y_i_)’s so that no two regions overlap.*

Using the above definitions, the problem of the multi-application mapping can be described as follows.

Given a set of applications where *S* = {*TG*_1_,*TG*_2_, … *TG_n_*} and an *NAG*, identify a mapping region *R_i_* on the *NAG* for each *TG_i_* which can allocate all tasks in *TG_i_* on the cores within the region *R_i_* such that the best performance is achieved. Then, identify the optimal placement *P* of the mapping regions for all the applications, such that the mapping area *A* formed by mapping regions for accommodating all applications is minimized, where *A* is typically measured by the final enclosing rectangle of *P*.

For an individual application mapping, the network performance—in terms of communication power consumption and the latency between task *t_i_* and *t_j_* executed in mapping cores in the *R_i_* of the NoC platform—is mainly determined by *v_i,j_* and the Manhattan distance *h_i,j_* between the mapping cores [4]. Hence, to achieve the best performance, we need to minimize the sum of the products of the *v_i,j_* and *h_i,j_* for all the communications in an application, which is to identify:

(1) $$\min\left\{\sum_{\forall a_{i,j}}v_{i,j}\times h_{i,j}\right\}$$

## 3. Multi-Application Mapping Algorithm

To achieve the goal of best network performance and minimize the mapping area of the NoC platform, we proposed a multi-phase-based multi-application mapping approach. The main procedure of our proposed algorithm is given in Algorithm 1. The mapping consists of three phases: Rectangle Analysis (RA), Task Mapping (TM) and Application Placement (AP). RA analyzes the potential mapping regions for a target single application under width and height bounds. TM is applied to different rectangles on the NoC platform to minimize network communication power consumption and latency, and then the rectangle which performs best is chosen as the mapping region for the application. AP is undertaken after TM to conduct the mapping of multiple applications, and to determine the optimal placement of chosen regions for multiple applications mapped on the NoC platform.
**Algorithm 1** The main procedure of the proposed multi-phase multi-application mappingInput: a set of *N* applications, a 2D Mesh based NoC architecture Output: mutli-application mapping results 1. for *i*=1 to *N* 2. analyze potential rectangles *R_i_*_1_, *R_i_*_2_, …*R_ij_*…, based on the number of tasks of the application and the bounds of width and height 3. for a single application, under different potential rectangles, a genetic algorithm is used to map tasks onto cores with selected regions on the NoC platform 4. identify the rectangle which performs best as the mapping region for the application 5. end for 6. packet each mapping region as a block and generate initial placement for multiple applications mapping using a B*Tree representation 7. use a simulated annealing algorithm to explore optimal placements 8. output the optimal solution

### 3.1. Rectangle Analysis

**Figure 1:**
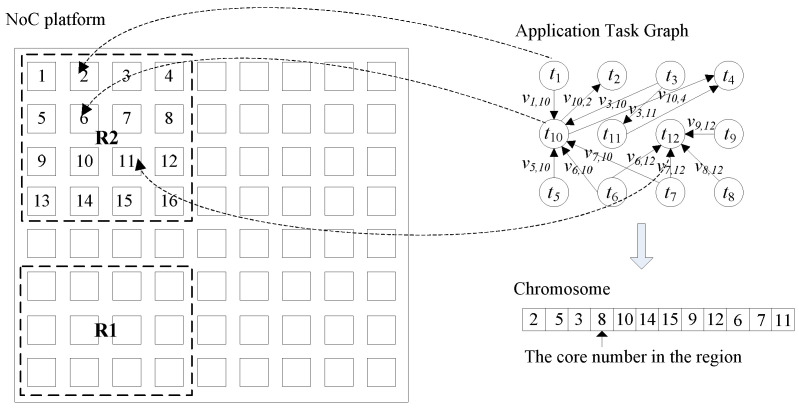
Potential mapping regions for an application with 12 tasks.

On a 2D Mesh NoC, any sub-mesh or rectangle can be regarded as a section of a compact area. Before task mapping, we needed to choose a rectangular region with a corresponding number of cores in an NoC platform for each individual application. To do this, we used an approximate-factorization solution. Given the number of tasks of an application and the rectangle bounds of width and height, we were able to generate several potential rectangles. Taking an application with 12 tasks as an example, the given width bound is 4, and the height bound is 4. Then we generate 4 × 3 (R1), 4 × 4 (R2)—two rectangles in an NoC platform—as potential regions for mapping as shown in Figure 1.

### 3.2. Task Mapping

In order to optimize network performance, an optimal mapping set is produced by utilizing a genetic algorithm (GA), which is similar to the single application mapping described in reference [9], and generally comprises four steps.

Firstly, an initial population of chromosomes is generated, which consists of many randomly generated task placements. Each chromosome is encoded into integer strings, with its length equal to the number of vertices in a TG, as shown in Figure 1. Then the fitness of each chromosome is evaluated in the second step. The fitness function here is given by Equation (1). In the third step, a new population is created by applying three operators (selection, crossover and mutation) similar to the natural selection operators. Finally, the optimal solution with minimized network power consumption and latency is selected at the end of a number of generations.

We used the above GA base mapping algorithm to map tasks of an application onto potential regions which can be obtained from the last phase. Then we compared the mapping results, and choose the region which performed best as the packeted region for the application.

### 3.3. Application Placement

**Figure 2:**
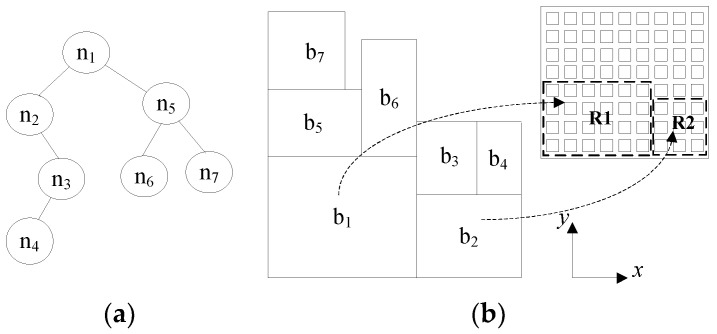
B*Tree structure and its corresponding placement: (**a**) B*-tree structure; (**b**) The corresponding placement.

After the packeted regions for each application were identified, we generated a layout for multi-application mapping regions utilizing B*Tree representation [16]. A B*-tree is an ordered binary tree for representing non-slicing floorplans. Given an admissible placement (in which no blocks can move left or down), a unique B*-tree can be constructed, which corresponds to a unique layout result. Figure 2 shows a B*-tree structure and its corresponding placement.

As shown in Figure 2, a unique placement of blocks can be generated through a given B*-tree structure, where node *n_i_* in the B*-tree represents a block *b_i_* placed in an NoC platform. The depth-first search (DFS) procedure is used to recursively traverse nodes in the B*-tree to generate the placement result. Starting from the root, the block corresponding to the root node is placed on the bottom-left corner and thus the coordinate of the block is (*x_root_*,*y_root_*) = (0,0). Then the left sub tree and right sub tree are recursively traversed, respectively. If node *n*_j_ is the left child of node *n_i_*, block *b_j_* is placed on the right-hand side and adjacent to block *b_i_*, i.e., *x_j_* = *x_i_* + *w_i_*, where *w_i_* represents the width of the block. Otherwise, if node *n_j_* is the right child of *n_i_*, block *b_j_* is placed above block *b_i_*, with the *x*-coordinate of *b_j_* equal to that of *b_i_*, i.e., *x_j_* = *x_i_*. Therefore, given a B*-tree, the *x*-coordinates of all blocks can be determined by traversing the tree once in linear time. Further, the *y*-coordinate of each block can be computed by a contour data structure related to the width and height of each block [17]. In our proposed approach, each block *b_i_* represents a mapping region for an application, as shown in Figure 2b.

**Figure 3:**
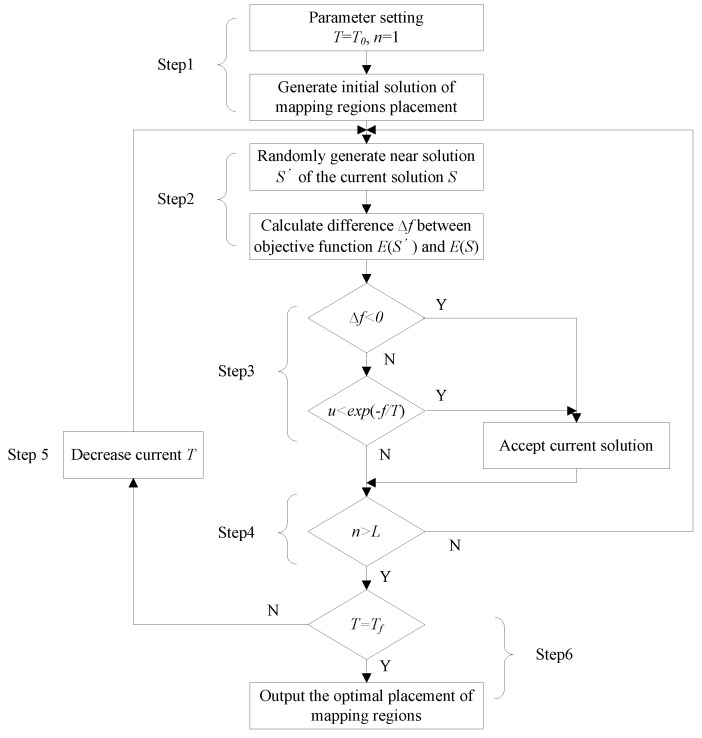
The optimal placement of the application mapping regions generated using SA.

Based on the B*-tree structure, we used SA to explore optimal placement solutions for multi-application mapping regions. SA is a global optimization search algorithm based on the physical principle of annealing. The algorithm flow used to generate an optimal placement using the B*-tree-based SA is shown in Figure 3.

Step 1: Set SA parameters, e.g., initial temperature *T* = *T*_0_, initial iteration time *n* = 1. Then generate an initial solution of regions placement using B*Tree representation, and calculate its corresponding objective function value *E*(*S*) represented by mapping area *A*.

Step 2: Based on the current solution *S*, a disturbance operation is adopted to generate a new solution *S*’, which contains three steps. First, rotate a block 90 degrees on the 2D plane. Second, swap the locations of the two nodes randomly selected in the B*Tree. Third, remove a node and insert it into a child node of its parent node randomly selected in the B*Tree. Afterwards, calculate the *E*(*S’*) of the new solution and difference Δf=E(S′)−E(S).

Step 3: Judge Δf, if Δf<0, accept *S*’ as the new current solution. Otherwise, accept *S*’ as the new current solution at a certain probability *exp*(−*f*/*T*).

Step 4: Under the current temperature *T*, repeat the disturbance and accept process (repeat Step 2 and Step 3) for *L* times.

Step 5: Decrease current temperature *T*=K_t*T, where *K_t* represents a cooling parameter.

Step 6: Judge whether current *T* is equal to the terminal temperature *T_f_*. If *T* = *T_f_*, output the optimal placement. Otherwise, return to Step 2.

## 4. Experimental Results

To verify the efficiency of our proposed multi-phase based multi-application mapping approach, we chose the approach described in [4] as a reference. Four benchmark applications were generated by TGFF with 25, 16, 16 and 9 tasks executed in a corresponding number of cores in an NoC platform [18]. Nirgam integrated with Orion was adopted as the NoC simulator to evaluate the network communication power consumption and latency [19,20]. The experimental parameters of the NoC architecture used in Nirgam are shown in Table 1. For the power results, the technology node was set to the default value of 110 nm in Nirgam.

**Figure 4:**
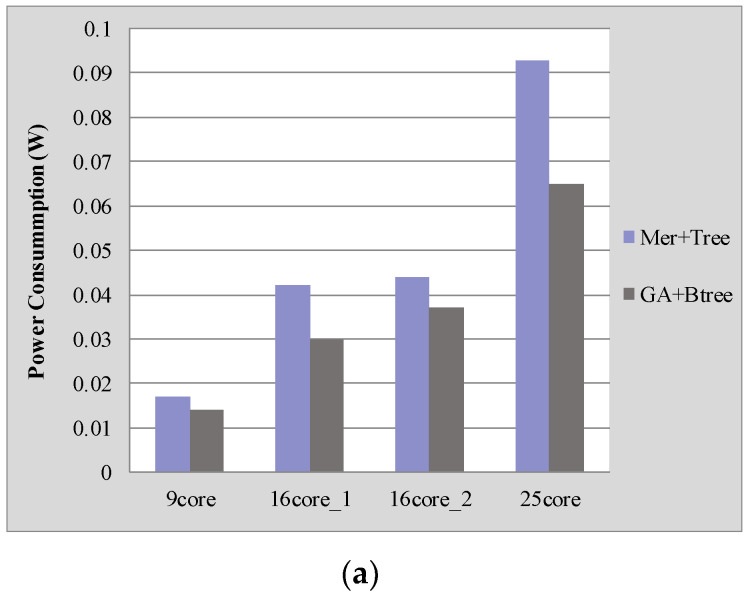

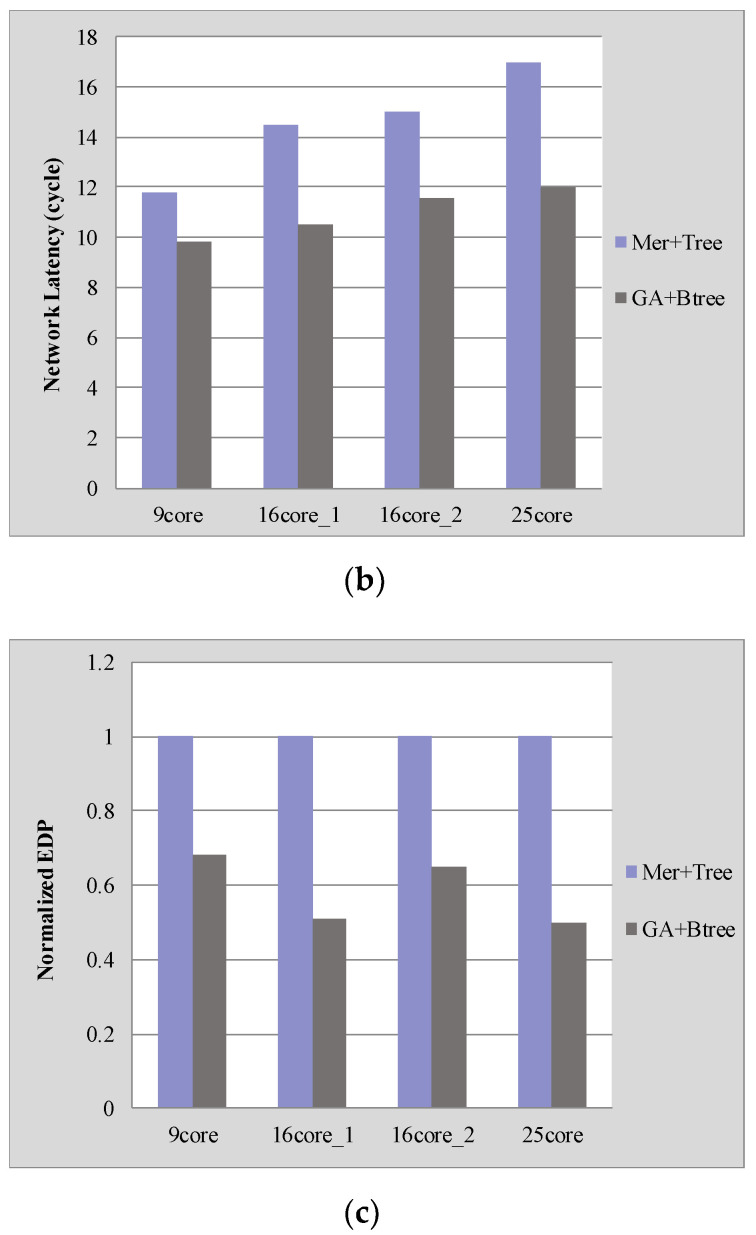
Performance comparison under different NoC platforms: (**a**) Power consumption comparisons; (**b**) Latency comparisons; (**c**) EDP comparisons.

Figure 4 shows the performance comparison between our proposed approach (GA + Btree) with the approach (Mer + Tree) described in [4] for four benchmarks. Our proposed approach saves on average 23.45% power consumption and 24.42% latency compared with the results generated with the approach described in [4].

We also analyzed the normalized energy-delay product (EDP) of different applications as shown in Figure 4c. The EDP with our approach of “GA + Btree” is much lower than that of the approach of “Mer + Tree”, which is reduced by about 42%. This is because our proposed approach reduces the communication distance between mapping cores, which is the main factor affecting the power consumption and delay of on-chip communication.

**Figure 5:**
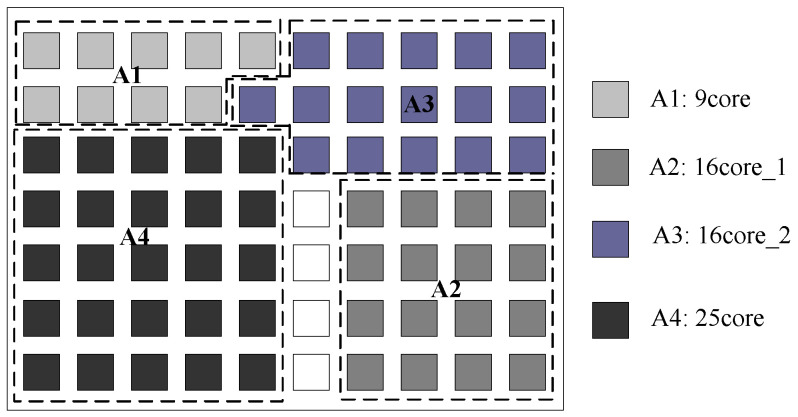
The placement generated by the approach in [4].

**Figure 6:**
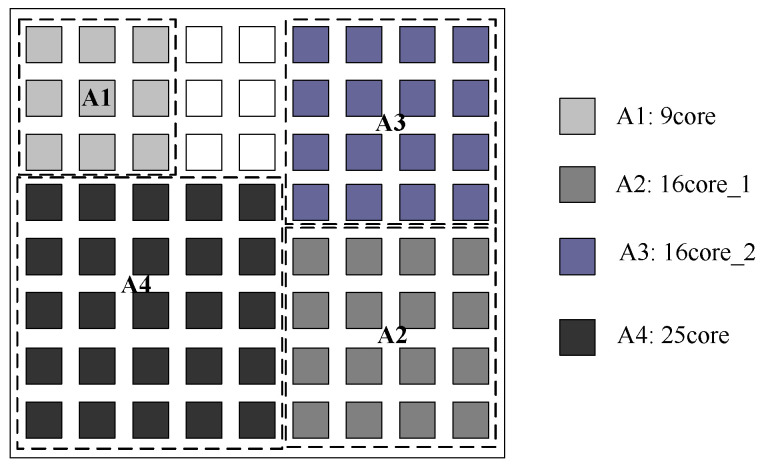
The placement generated by our approach.

Figure 5 shows the placement result of reference [4] which takes the Mer technique and tree model algorithm under a fixed 10 × 7 NoC platform [4]. Figure 6 shows the result of our proposed approach. Our proposed approach first considers each application mapping separately, and then generates the placement of multiple application mapping regions to determine the optimal scale of the NoC platform. The NoC platform generated by our proposed approach is 9 × 8. Hence, our approach may have more blank spaces, but it secures better performance.

**Figure 7:**
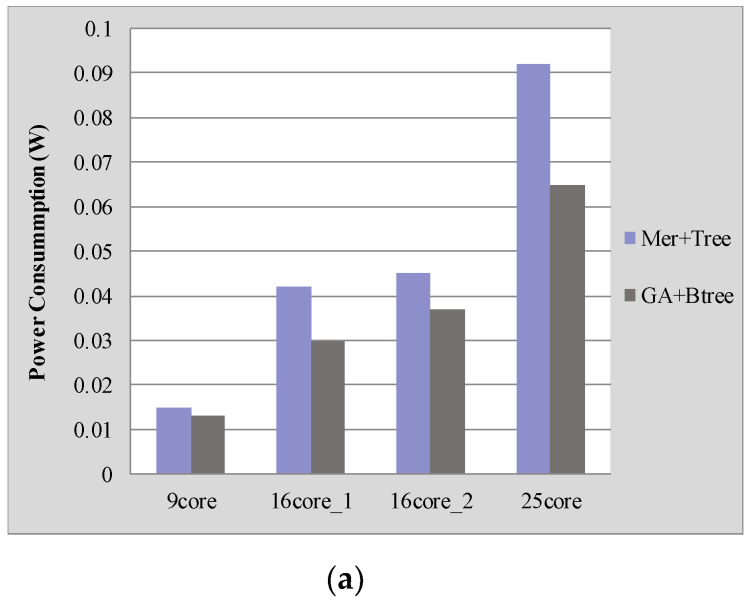

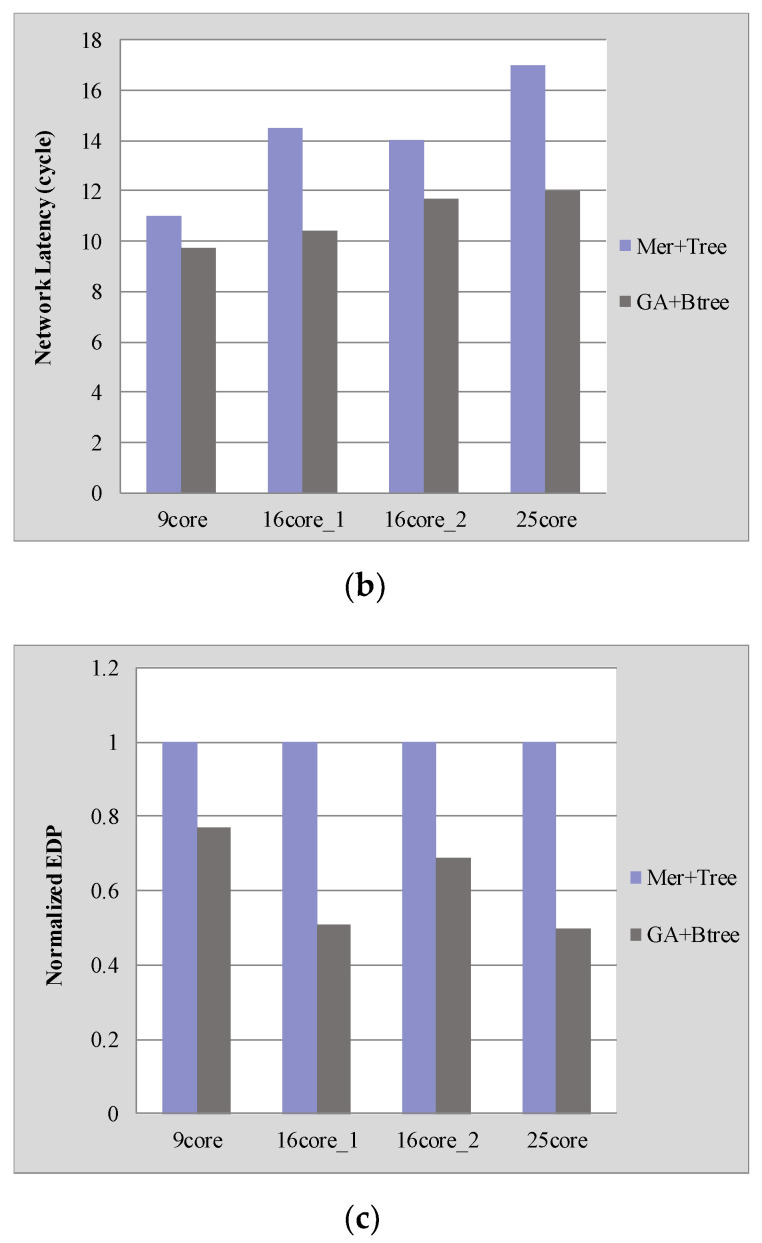
Comparison of performances under the same NoC platform: (**a**) Power consumption comparisons; (**b**) Latency comparisons; (**c**) EDP comparisons.

Furthermore, we compared our proposed approach with the approach in [4] under the same NoC platform. The performance comparison is shown in Figure 7. Our proposed approach saved 21.92% power consumption and 21.85% latency on average compared with the result described by the author of [4], and the EDP with our approach of “GA+Btree” is also much lower than that of the approach of “Mer+Tree” described in [4], which was reduced by about 38%.

## 5. Conclusions

In this paper, we proposed a multi-phase-based multi-application mapping approach for NoC design. In the first phase, our approach started with rectangle analysis which identified several potential regions for an application. In the second phase, a GA based mapping algorithm was used to map all tasks of the individual application into these potential regions and identify the one which exhibited the strongest performance. In the third phase, each application mapping region was packeted as a region, and a B*Tree based SA algorithm was used to generate the optimal placement for multi-application mapping regions in an NoC platform. The experiment results show that, compared with existing multi-application mapping schemes, the proposed approach can achieve considerable reduction of network power consumption (up to 23.45%) and latency (up to 24.42%) for a given set of applications.

## Figures and Tables

**Table 1:** The experimental parameters of the NoC architecture.

Parameter Name	Value
Topology	10 × 7 Mesh, 9 × 8 Mesh
Routing algorithm	XY
Packet size (flit)	2
Router buffer (flit)	5
Clock frequency (GHz)	1

## Data Availability

Not applicable.
